# Acetylsalicylic Acid Reduces the Severity of Dextran Sodium Sulfate-Induced Colitis and Increases the Formation of Anti-Inflammatory Lipid Mediators

**DOI:** 10.1155/2013/748160

**Published:** 2013-09-08

**Authors:** Thomas Köhnke, Beate Gomolka, Süleyman Bilal, Xiangzhi Zhou, Yanping Sun, Michael Rothe, Daniel C. Baumgart, Karsten H. Weylandt

**Affiliations:** ^1^Department of Gastroenterology, Hepatology and Endocrinology, Virchow-Hospital, Charité Medical School, Free and Humboldt-University of Berlin, 13353 Berlin, Germany; ^2^Laboratory for Lipid Medicine and Technology, Massachusetts General Hospital and Harvard Medical School, Boston, MA 02114, USA; ^3^Department of Radiology, Brigham and Women's Hospital, Harvard Medical School, Boston, MA 02115, USA; ^4^Lurie Family Imaging Center, Dana-Farber Cancer Institute and Harvard Medical School, Boston, MA 02115, USA; ^5^Lipidomix GmbH, 13125 Berlin, Germany

## Abstract

The role of non-steroidal anti-inflammatory drugs in inflammatory bowel disease is controversial, as they have been implicated in disease aggravation. Different from other cyclooxygenase inhibitors, acetylsalicylic acid (ASA) enhances the formation of anti-inflammatory and proresolution lipoxins derived from arachidonic acid as well as resolvins from omega-3 polyunsaturated fatty acids such as docosahexaenoic acid (DHA). In this study, we examined the effect of ASA on murine dextran sodium sulfate colitis. A mouse magnetic resonance imaging (MRI) protocol and post mortem assessment were used to assess disease severity, and lipid metabolites were measured using liquid chromatography-coupled tandem mass spectrometry. Decreased colitis activity was demonstrated by phenotype and MRI assessment in mice treated with ASA, and confirmed in postmortem analysis. Analysis of lipid mediators showed sustained formation of lipoxin A4 and an increase of DHA-derived 17-hydroxydocosahexaenoic acid (17-HDHA) after treatment with ASA. Furthermore, *in vitro* experiments in RAW264.7 murine macrophages demonstrated significantly increased phagocytosis activity after incubation with 17-HDHA, supporting its proresolution effect. These results show a protective effect of ASA in a murine colitis model and could give a rationale for a careful reassessment of ASA therapy in patients with inflammatory bowel disease and particularly ulcerative colitis, possibly combined with DHA supplementation.

## 1. Introduction

The inflammatory bowel diseases (IBDs) ulcerative colitis (UC), confined to the colon, and Crohn's disease (CD), affecting the whole gastrointestinal tract, are chronic inflammatory disorders with considerable morbidity and, particularly for UC, mortality due to a high risk of colorectal cancer development. Current therapy for IBD focuses on drugs that inhibit inflammation [1–4]. Human data regarding the potential benefits and risks of COX inhibition in patients with IBD are conflicting [5–9]. This is also reflected in animal studies examining COX inhibition in experimental colitis models. While some recent studies found deleterious effects in animals with DSS colitis associated with inhibition of COX [10–13], others demonstrated a benefit of COX-2 inhibitor treatment [14, 15]. 

Recently, newly discovered lipid mediators have been implicated in the alleviation of colitis [16]. The lipoxins are lipid mediators originating from the omega-6 polyunsaturated fatty acid (n-6 PUFA) arachidonic acid (AA) by enzymatic action of different lipoxygenases or by acetylated COX-2 [17]. Studies have demonstrated potent anti-inflammatory and inflammation dampening properties for lipoxins [18], and stable analogues of lipoxins were shown to alleviate experimental colitis [19, 20]. Similarly, protective lipid mediators (so-called resolvins) can be formed from omega-3 polyunsaturated fatty acids (n-3 PUFAs) such as eicosapentaenoic acid (EPA) and docosahexaenoic acid (DHA). Our previous work demonstrated formation of resolvins in mice with increased omega-3 fatty acid tissue content in the context of DSS colitis [21], and other studies have shown an anti-inflammatory effect of several of these compounds in the context of experimental colitis models [22–24].

In this study, we analyzed the effect of acetylsalicylic acid (ASA) in chemically induced acute DSS colitis in mice. DSS colitis is characterized by initial injury to the epithelial cells followed by inflammation and has been shown to be a good model to test therapeutics for human inflammatory bowel disease [25]. Many studies have used this model in the past as it is regarded as a good animal model of IBD, and particularly UC, with a predominant site of inflammation in the distal colon [26].

To evaluate disease activity in the different groups, we extended our analysis beyond the conventional phenotype and morphology markers and employed small animal MRI technology. Noninvasive imaging in murine models of colitis has been previously described using CT and MRI technologies [27–29]. For the study presented here, a modified protocol was devised in order to reliably measure and compare colon wall thickness and hyperemia in different mice. 

To assess possible mechanisms responsible for the observed protection in ASA-treated mice, mass spectrometric measurements were then employed to determine hydroxylated lipid metabolites and mediators derived from AA (lipoxin A_4_ and 15-epi-lipoxin A_4_) and from 17-hydroxydocosahexaenoic acid (DHA). 

## 2. Materials and Methods

### 2.1. Induction of Colitis

Female C57BL/6 mice were obtained from Charles River Laboratories (Wilmington, MA) and held until the desired age (6 weeks) and body weight (19–21 g). 

For testing of different treatment regimens, mice were divided into 3 groups. One group received 3% DSS dissolved in sterile drinking water and daily intraperitoneal (ip) injections of 0.5 mg acetylsalicylic acid (ASA, Sigma-Aldrich, St. Louis, MO) dissolved in 300 *μ*L of vehicle (3.33% EtOH in 0.9% NaCl) for 5 days (*n* = 5). Another group received 3% DSS as well as ip injections of the vehicle alone for 5 days (*n* = 6). Finally, the last group received sterile drinking water and ip injection of vehicle alone and served as a control (*n* = 3). The first ip injection of ASA or vehicle was administered right before the mice were switched to DSS-containing drinking water. After 5 days, the DSS-containing water was replaced with sterile drinking water. 

### 2.2. Evaluation of Colitis Severity

Body weight measurement (presented as percentage of body weight on day 0) as well as evaluation of stool status was carried out daily. Stool samples from individual mice were evaluated on a three-point scale using a test for occult blood (Hemoccult, Beckman Coulter Inc., Fullerton, CA) as well as macroscopic evaluation (0 = no occult blood; 1 = test for occult blood slightly positive; 2 = test for occult blood strongly positive; and 3 = bloody stool). On day 9, mice were sacrificed, colons were excised, and length was measured. All samples were snap frozen in liquid nitrogen and stored at −80°C for further analysis.

### 2.3. Histological Evaluation

Frozen tissue sections from the distal colon of all mice were mounted in OCT medium. Sections were cut at 5 *μ*m thickness and mounted on glass slides. After air drying, sections were fixed in fixing medium (70% ethanol, 4% formaldehyde, and 5% glacial acetic acid in H_2_O), washed in ethanol (70%) and water, followed by standard hematoxylin and eosin stain. Slides were evaluated in a blinded manner and scored according to severity of inflammation (0 = no inflammation, 1 = mild, 2 = moderate, and 3 = severe), thickness of inflammatory involvement (0 = no inflammation, 1 = mucosa, 2 = mucosa plus submucosa, and 3 = transmural), character of epithelial damage (0 = intact epithelium, 1 = disruption of architectural structure, 2 = erosion, and 3 = ulceration), and extent of lesions (0 = no lesions, 1 = punctuate, 2 = multifocal, and 3 = diffuse). This evaluation protocol is based on a previously described scoring system for DSS colitis [30].

### 2.4. Magnetic Resonance Imaging (MRI)

**Figure 1:**
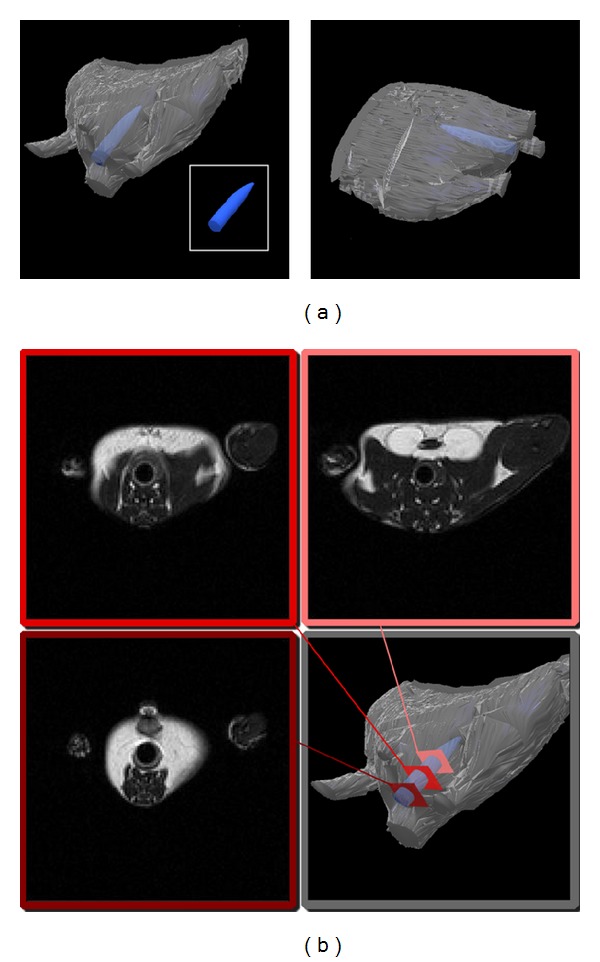
MRI analysis of colitis severity. (a) For MRI analysis of disease severity, a plastic tube with outer diameter of 2.1 mm was carefully placed approximately 9 mm into the rectum of the anaesthetized mouse. (b) Analysis was performed by comparing wall thickness as well as brightness between the different mice in three defined slices.

Mice were scanned on day 3 and day 8 of the experimental protocol. MRI assessment of colitis was performed using a Bruker 4.7T Avance horizontal bore system (Bruker Avance, Karlsruhe, Germany) equipped with a 200 mm gradient set capable of 30 G/cm gradient strength. During the MR scan, the mice were mounted on a home-built nonmagnetic holding bar equipped with a nose cone for anesthesia delivery and were anaesthetized using 1.5%–2% (vol/vol) isoflurane (Baxter, Deerfield, IL) in 1 L/min oxygen flow. The breathing rate of the mice was monitored using a small animal monitoring and gating system (Small Animal Instruments Inc., NY). The isoflurane flow was adjusted to achieve a breathing rate of 20–30 breaths per minute during the scanning process. In order to standardize the comparisons of wall thickness as well as colon wall signal intensity between different animals, a nonmagnetic (plastic) tube (outer diameter 2.1 mm) was placed approximately 9 mm into the rectum of the anaesthetized mice (Figure 1(a)). For each mouse, 24 T2 weighted 2D axial images were acquired to cover the colon using a fast spin echo sequence with a repetition time (TR) of 2683 ms, an echo time (TE) of 12 ms, a 128 × 128 matrix, and a 3.0 cm × 3.0 cm field of view (FOV). The slice thickness was set to 1 mm, and the number of averages was 8. After image acquisition, the plastic tube was removed, and the animals were transferred to a heating pad until they were fully awake. The images were analyzed using standard imaging software (Adobe Photoshop, Adobe Systems, San Jose, CA).

Colon wall thickness was then measured on the T2 weighted images from three slices of the distal colon with a standardized distance of 3 mm between slices (Figure 1(b)). For each slice, three measurements were performed, and the means for each animal were calculated. For signal intensity analysis, a region of interest (ROI) was drawn within the colon wall. A histogram tool was used to analyze the brightness of the pixels within the ROI. This was repeated for the same three slices that were used for the wall thickness measurements. The pixel brightness means were then normalized by comparison with the nearby fatty tissue around the ovaries of the mice.

### 2.5. Lipid Metabolite Analysis

30 mg ground and frozen colon tissue was mixed with 500 *μ*L water and 500 *μ*L methanol, internal standard consisting of 10 ng LTB_4_-d_4_ was added, and the sample was then hydrolyzed with 300 *μ*L of 10 M sodium hydroxide for 30 minutes at 60°C. The solution was neutralized with 60% acetic acid, and the pH was adjusted to 6.0 with sodium acetate buffer. A solid phase extraction was performed as previously described [31]. For elution, an n-hexane:ethyl acetate extraction mixture (25 : 75) with 1% acetic acid was used. The eluate was evaporated on a heating block at 40°C under a stream of nitrogen to obtain a solid residue. Residues were then dissolved in 70 *μ*L acetonitrile and measured using an Agilent 1200 HPLC system coupled to an Agilent 6410 Triple quad mass spectrometer with an electrospray ionization source. Analysis of lipid metabolites was performed with multiple reaction monitoring in negative mode. 

### 2.6. Phagocytosis Assay

RAW 264.7 cells were grown in Dulbecco's modified Eagle's medium (DMEM) supplemented with 10% fetal bovine serum, 100 U/mL penicillin, and 100 U/mL streptomycin. All experiments were performed in a humidified atmosphere under 5% CO_2_ at 37°C. Cells were plated in 96-well plates at a density of 1 × 10^5^ cells/well and incubated overnight. To quantify phagocytosis, cells were incubated with fluorescein-labelled bioparticles (Vybrant Phagocytosis Assay, Invitrogen, Carlsbad, CA), diluted 1 : 2. Uptake was measured after 4 h in a fluorescence plate reader, essentially following the manufacturer's protocol. To evaluate the effect of 17-HDHA on phagocytosis, cells were pretreated with 1 *μ*M 17-HDHA for 2 h. Positive controls with equivalent volumes of ethanol (vehicle) and negative controls were performed in parallel.

### 2.7. Statistical Analysis

Statistical analysis was performed with GraphPad Prism 3.02v Software (GraphPad, La Jolla, CA). Comparison was made using the Mann-Whitney *U* test or as indicated. All values are presented as the mean ± SEM. Statistical significance was set at *P* < 0.05.

## 3. Results

**Figure 2:**
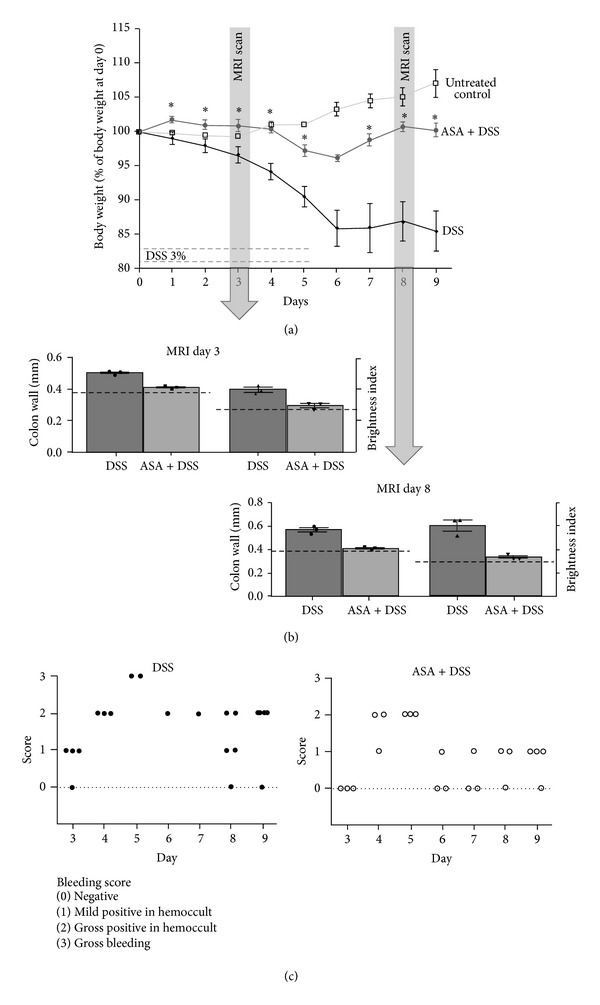
*In vivo* assessment of colitis severity. (a) Body weight course in the different groups, *n* = 3 for the control group, *n* = 5 for the ASA-group, and *n* = 6 for the DSS group; *P < 0.05 versus DSS. (b) MRI-measured colon wall thickness and brightness on day 3 as well as day 8 of the experimental protocol, *n* = 3 for each treatment group. In dashed lines, the values of measurements in untreated control mice are shown. (c) Bleeding score evaluation in animals treated with DSS or ASA + DSS. There was no bleeding in control animals (not shown).

Induction of colitis resulted in marked changes in body weight, appearance of fecal blood, and general status. Mice treated with ASA showed significantly less body weight loss (Figure 2(a)) as well as a recovery of body weight within 3 days after the end of DSS exposure, while untreated mice exhibited only a stabilization of body weight after cessation of DSS. 

Colitis disease activity was evaluated by MRI on day 3 and day 8 of the treatment protocol. The MRI results show a marked difference between the treated groups of mice with less wall thickness and a significantly lower T2 weighted signal intensity (brightness) in the mice treated with ASA, as signs of a lower inflammatory infiltration (Figure 2(b)). Due to technical limitations in the number of MRI scans we were able to perform, only 3 animals per group were assayed, which does not give enough values for statistical normality testing using, for example, the Kolmogorov-Smirnov test. Applying nonparametric Mann-Whitney *U* testing to these data led to nonsignificant findings. However, when hypothesizing normal distribution—and thus applying Student's *t*-test—for these data, the differences for thickness as well as brightness were significant on day 3 and day 8 with *P* < 0.01 when comparing the ASA + DSS group versus DSS alone.

The results of the MRI and body weight analyses confirmed the decreased disease activity in the mice treated with ASA. Furthermore, assessment of bloody stools in the animals showed a trend towards more severe and prolonged bleeding in the DSS group (Figure 2(c)). However, conclusive statistical evaluation of these data could not be performed, as it was not possible to do stool testing on every animal every day of the experimental protocol.

**Figure 3:**
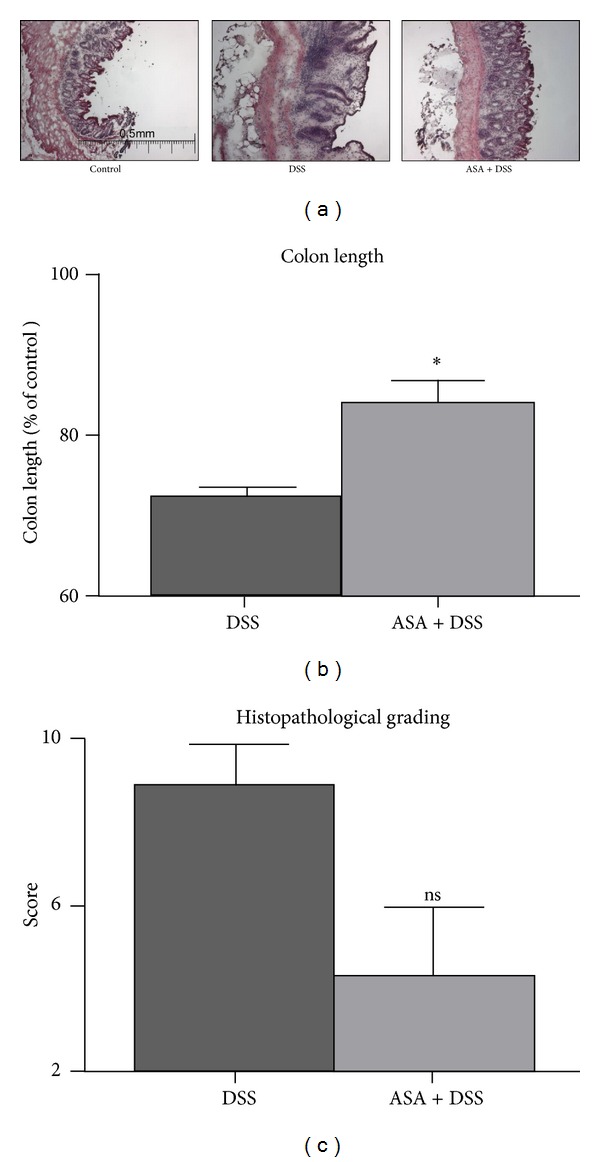
Postmortem assessment of colitis severity. (a) Hematoxylin and eosin stains of transmural colon walls. Representative images of control tissue as compared to animals treated with DSS and ASA + DSS. (b) Colon shortening as an indicator of disease severity was compared between the different experimental groups, *n* = 5 for the ASA-group and *n* = 6 for the DSS group; *P = 0.044 versus DSS. (c) Histological grading score, *n* = 5 for the ASA-group and *n* = 6 for the DSS group; ^ns^ *P* = 0.054 versus DSS. Untreated controls (not shown) reached a maximum of 2 points in this score.

The *in vivo* disease activity assessments were validated in the postmortem analysis of the colons, with wall thicknesses similar to those observed in the MRI measurements (Figure 3(a)) and significantly less colon shortening in mice treated with ASA as compared to mice exposed only to DSS (Figure 3(b)). Microscopic grading of the changes in the colon revealed decreased inflammatory activity in mice treated with ASA (summarized scores are shown in Figure 3(c)). However, we were not able to document changes in inflammatory activity biochemically, as serum TNF-*α* levels, measured by ELISA, were not significantly different between the different treatment groups (data not shown).

**Figure 4:**
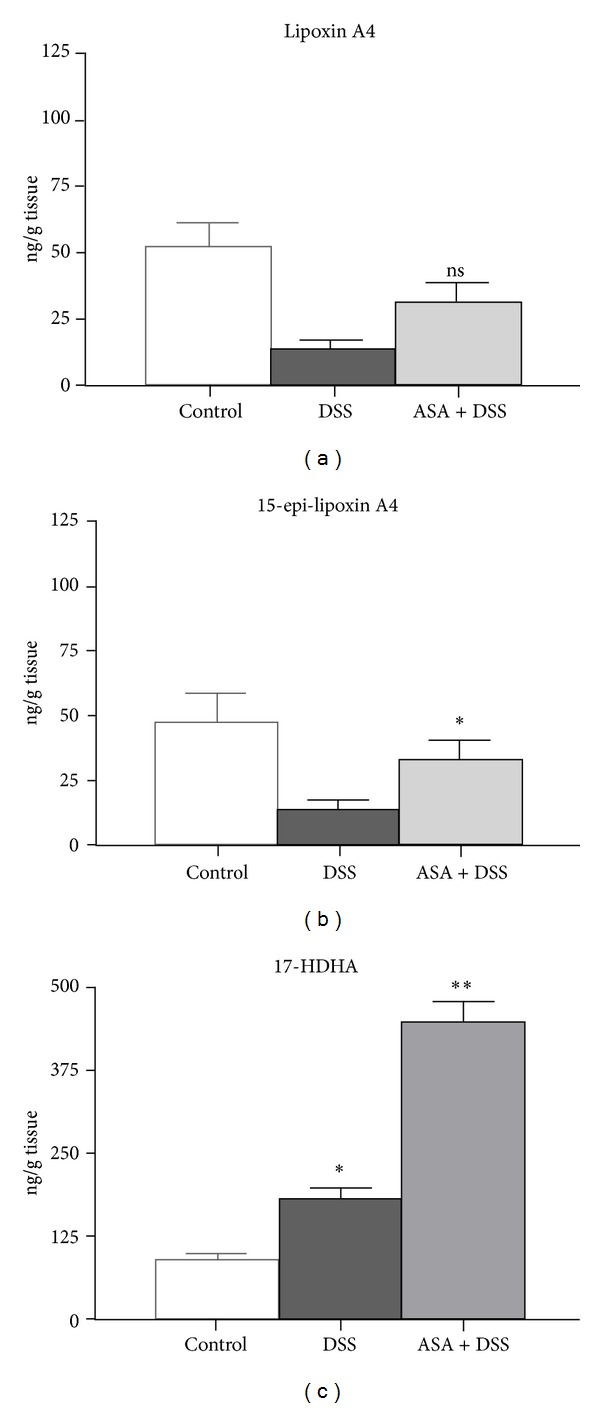
Assessment of lipid mediators. Shown are the arachidonic acid-derived lipoxin A_4_ (a) and 15-epi-lipoxin A_4_ (b) with *n* = 3 for the control group, *n* = 5 for both the DSS alone and the DSS plus ASA groups; ^ns^ *P* = 0.056 versus DSS (not significant); *P = 0.016 versus DSS. (c) The levels of 17-HDHA, with *n* = 3 for the control group, *n* = 5 for both the DSS alone and the DSS plus ASA groups; *P = 0.036 versus controls; **P = 0.008 versus DSS (b).

As lipid mediators formation of AA-derived lipoxins and n-3 PUFA-derived resolvins can be increased by ASA-induced acetylation of the COX-2 enzyme [32], we assessed several of these mediators and their precursors in the colons of mice with DSS-induced colitis with and without treatment with ASA. Due to the presence of AA and DHA but not EPA in the colon tissue of the mice used in this study (data not shown), we focused on the assessment of AA-derived lipoxins and DHA-derived 17-hydroxydocosahexaenoic acid (17-HDHA). Lipoxin A_4_ and 15-epi-lipoxin A_4_ concentrations were decreased in colon tissue from mice three days after DSS exposure, but formation of 15-epi-lipoxin A_4_ was significantly higher in mice treated with ASA as compared to the DSS alone treatment group (Figures 4(a) and 4(b)). Unexpectedly, the DHA-metabolite 17-HDHA was significantly higher in colon tissues from mice three days after DSS exposure (Figure 4(c)) and was increased even more in tissue samples from mice treated with ASA. 

**Figure 5:**
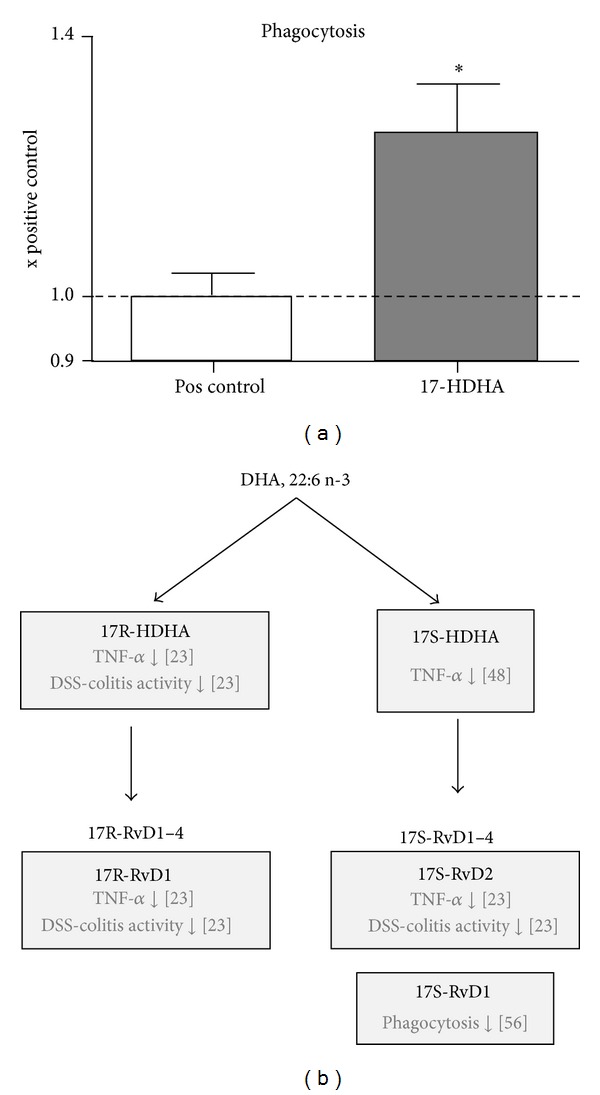
Effect of 17-HDHA on phagocytosis and overview of DHA-derived lipid mediators. (a) Effect of 17R/S-HDHA on phagocytosis in RAW 264.7 murine macrophages (*n* = 12, *P = 0.026). (b) Synthesis pathways of DHA-derived anti-inflammatory lipid mediators arising from 17R- and 17S-HDHA, and the resulting mediators are called the resolvins D1-4. Included also are the references to the published effects of the DHA-derived mediators on TNF-*α* secretion from macrophages [23, 48], phagocytosis [56], and DSS-colitis activity [23].

In order to further elucidate the mechanism by which 17-HDHA might contribute to inflammation dampening and resolution, the *in vitro* ability of 17-HDHA to increase phagocytosis was tested. In these experiments, an assay system with fluorescently labeled *E. coli* K-12 bioparticles was used as a tool to study overall phagocytotic activity of macrophages [33, 34]. A significant (1.25-fold) increase in phagocytosis activity after 1 *μ*M 17-HDHA preincubation in the RAW 264.7 murine macrophage cell line was demonstrated (Figure 5(a)).

## 4. Discussion

The data presented here indicate that DSS-induced colon inflammation can be alleviated by acetylsalicylic acid. Although a number of previous studies have examined the effectiveness of COX inhibitors in the context of DSS colitis, the outcomes of these studies were inconsistent or conflicting [10–15], and, to our knowledge, ASA was never used in experimental DSS colitis. This may be due to the fact that ASA causes ulcerations, erosions, and gastrointestinal bleeding in the stomach as well as the small bowel, and many clinicians believe it therefore to be too GI toxic to be of any value in the control of colonic inflammation. 

At the same time, patients with IBD, and particularly UC, remain at increased risk for colorectal cancer [35, 36]. Long-term ASA treatment has been shown to be effective for colon cancer prevention [37–40]. In patients with UC, although data in this field is scarce, a similar risk reduction of colorectal cancer could be shown [41], arguing for ASA use also in these patients. 

We set out to study the effect of ASA supplementation on the course of DSS-induced colitis in mice, hypothesizing an effect of aspirin treatment on the formation of hydroxylated lipid mediators such as the lipoxins as well as DHA-derived compounds. These were characterized as potent anti-inflammatory compounds also in the context of DSS colitis in previous studies [19, 23]. 

In our experiments, we aimed for an ASA dose comparable to low-dose ASA administration in humans. Previously published data on low-dose ASA administration in mice with intestinal or other tumors used doses of 40 mg/kg [42] and 50 mg/kg [43]. High-dose ASA dosage was at 400 mg/kg in a previous tumor prevention study in mice [44]. Two studies in intestinal tumor models used a dose of 25 mg/kg [45, 46], which the authors calculated to correspond to a dose of 80–110 mg/day in humans on a 2000 kcal diet following nutrient density calculations [47], and we decided to use this dose in our experiment to mimic low-dose ASA administration. A limitation of the data presented here is that no experiments with ASA only treatment were performed. However, the previous study by Mahmoud et al. has established the safety of long-term administration of ASA at a dose of 25 mg/kg in mice [45]. 

In the study presented here, we found significant changes in the tissue concentrations of anti-inflammatory lipoxins, with suppression of lipoxin A_4_ and 15-epi-lipoxin A_4_ in the mice with DSS colitis. As compared to this, the lipoxin levels in the ASA-treated animals were higher, which could contribute to the protection from colitis seen in these animals. In contrast, the amount of the DHA-derived 17-HDHA increased in animals after DSS treatment and even more so with concomitant ASA treatment. While higher levels of 17-HDHA were present in the colons from mice after DSS colitis induction as compared to controls, the treatment with ASA led to even higher 17-HDHA tissue concentrations. 

17-HDHA is the precursor for the formation of anti-inflammatory D resolvins, and several previous studies from other groups have demonstrated biological effects of the different stereoisomers of this compound, as summarized in (Figure 5(b)). It was shown that TNF-*α* secretion from murine macrophages can be suppressed by 17S-HDHA [48]. Furthermore, it has been demonstrated that 17S-HDHA is a precursor of the D(S)-resolvins, which are potent anti-inflammatory mediators [49–51]. Similarly, the 17-HDHA epimer 17R-HDHA as well as the D(R)-resolvins were recently shown to be anti-inflammatory in the context of experimental colitis, peritonitis, and arthritis [23, 51, 52]. 

The liquid chromatograph tandem mass spectrometry (LC-MS/MS) setup used in the study presented here was not able to differentiate between the 17S- and 17R-HDHA epimers and could not reliably identify resolvin D1 in tissue samples. However, as both epimers of 17-HDHA are precursors of anti-inflammatory D-resolvins, the increase in total 17(R/S)-HDHA observed in this study is an indicator of significantly increased formation of these anti-inflammatory lipid mediators derived from DHA.

Indeed, our *in vitro* results presented previously showed a 17-HDHA-triggered decrease in TNF-*α* secretion [53]. Together with the increased phagocytosis activity by 17-HDHA shown here (Figure 5), this indicates that a mix of the 17R/S-HDHA epimers itself carries anti-inflammatory activity. Data for lipoxin A_4_ [54, 55] and resolvin D1 [56] have established the concept of increased phagocytosis as a marker of inflammation dampening and resolution. Therefore, the data shown here for 17-HDHA add to the hypothesis that increasing the phagocytosis activity of macrophages is an important effect of the hydroxylated anti-inflammatory and proresolution lipid mediators derived from either AA or DHA.

Concerning the anti-inflammatory effect of ASA, the results presented here support additional anti-inflammatory mechanisms of ASA as compared to the mere inhibition of prostaglandin synthesis, by enhancing the formation of 17-HDHA in murine colitis. Together with the established effect of ASA in colon cancer prevention, the findings presented here might thus be able to contribute to interest in a careful reevaluation of ASA therapy in patients with UC with its high risk of colon cancer development, possibly in combination with DHA supplementation.
